# Histopathologic and molecular diagnosis of gastrointestinal tuberculosis in an immunocompetent patient: case report

**DOI:** 10.1128/asmcr.00105-24

**Published:** 2025-07-03

**Authors:** Sashank Cherukuri, Emily B. Huang, Arthur H. Totten, Jonathan Steinberg, Sunjida Ahmed

**Affiliations:** 1 Stony Brook University Hospital 22161, Stony Brook, New York, USA; Pattern Bioscience, Austin, Texas, USA

**Keywords:** *Mycobacterium*, Tuberculosis, gastrointestinal, immunocompetent, histopathology

## Abstract

**Background:**

Gastrointestinal tuberculosis (GI TB) is an extrapulmonary manifestation of infection with the bacterium *Mycobacterium tuberculosis*. While GI TB was once considered a rare entity, it has increasing prevalence with the use of immunosuppressive treatments. Diagnosis can be challenging as GI TB can be misdiagnosed as other primary etiologies.

**Case Summary:**

Here, we present a case of GI TB in which the patient was immunocompetent and presented with nonspecific symptoms, and an interferon-gamma release assay, which was positive. Subsequent biopsies were performed of the ascending colon, all showing positivity on acid-fast bacilli (AFB, Ziehl-Neelsen) and Fite stains, along with non-caseating granulomas on histopathologic review.

**Conclusion:**

In the presented case, the patient is immunocompetent. GI TB was diagnosed after histopathologic examination of colonic biopsies. This highlights the importance of having a high index of suspicion for unusual presentations of tuberculosis, especially in patients who come from endemic areas or who might have been exposed recently.

## INTRODUCTION

Tuberculosis (TB) is a major global health concern. According to the World Health Organization, it is one of the top 10 causes of death worldwide with an incidence of over 10 million cases each year in recent years (https://www.who.int/news-room/fact-sheets/detail/the-top-10-causes-of-death, https://www.who.int/teams/global-programme-on-tuberculosis-and-lung-health/tb-reports/global-tuberculosis-report-2024/tb-disease-burden/1-1-tb-incidence). Caused by the organisms of the *Mycobacterium tuberculosis* complex (MTB), TB most commonly affects the lungs but may have extrapulmonary manifestations in other organ systems (1, 2). Considering the wide variety of manifestations of extrapulmonary TB, this report will concentrate specifically on gastrointestinal tuberculosis (GI TB). A distinction must be made between abdominal TB, which encompasses the organs and structures within the abdominal cavity (affecting the lymph nodes, peritoneum, vessels, etc.), and GI TB, which affects organs in the gastrointestinal system (such as the mouth, esophagus, stomach, liver, biliary tree, intestines, rectum, and anus) (3).

GI TB can be mistaken for other disease processes due to the chronicity of infection. It is known as “the great mimicker” and can masquerade as a number of disease processes, including Crohn’s disease, intestinal sarcoidosis, and acute infections, such as appendicitis, colitis, acute cholecystitis, and necrotizing fasciitis (4). It is especially important to distinguish between GI TB and Crohn’s disease because initiation of immunosuppressants in a patient with TB increases the risk of extrapulmonary dissemination (5).

Routes of TB acquisition vary, especially with regard to GI TB. Direct ingestion, in which the sputum containing TB bacterium is coughed out of the lung and subsequently swallowed, is one route. Others include lympho-hematogenous dissemination, contiguous spread from adjacent organs or lymph nodes, and, though uncommon, through ingestion of contaminated, unpasteurized dairy products (6). The progression of disease may cause the formation of ulcers, strictures, or fistulas (6). Usually, granulomatous inflammation is present on histopathologic analysis (6).

## CASE PRESENTATION

The patient is a 29-year-old male with no significant past medical history, who tested negative for human immunodeficiency virus (HIV), who presented to the Stony Brook University Hospital (SBUH) Emergency Department with a 2-month history of right lower quadrant (RLQ) abdominal pain. Initially, he reported episodic mild-to-moderate post-prandial pain, which subsequently progressed to constant, severe RLQ and epigastric pain. The pain was associated with loose and infrequent blood-streaked bowel movements as well as nausea and decreased appetite. Review of systems identified a 20-lb unintentional weight loss, non-productive cough, chills, headaches, and dizziness. He denied any fever, rhinorrhea, chest pain, or dyspnea.

He is from Ecuador and had been living in the United States for 5 years. He had not previously received the Bacille Calmette-Guérin (BCG) vaccine and denied any prior TB exposure. He had no personal or family history of gastrointestinal (GI) infections or inflammatory bowel disease. He had no prior endoscopies or colonoscopies. Per the patient, he had been previously evaluated at other hospitals where blood work, stool studies, and an abdominal ultrasound were unremarkable. He had been empirically treated with antacid medications without improvement.

On presentation, vitals were notable for tachycardia (heart rate of 124 bpm) and hypotension (blood pressure of 90/59 mmHg); he was afebrile had a normal respiratory rate, and his oxygen saturation was normal on room air. Initial blood chemistries, hepatic panel, coagulation levels, and lipase levels were within normal limits. Complete blood count revealed borderline anemia (hemoglobin 13.1 g/dL, range 12.4–16.8 g/dL) with microcytosis (mean corpuscular volume of 77.7 fL, range 81.0–100.0 fL), and thrombocytosis (platelet count 408 K/μL, range 150–400 K/μL). A QuantiFERON TB (QFT, Qiagen) was positive. Sputum Gram stain completed in combination with routine lower respiratory bacterial culture revealed Gram-positive cocci and Gram-variable rods, but culture grew only normal flora. Stool studies, including ova and parasite, Gram stain, and GI pathogens panel (bioMérieux) were all negative.

**Fig 1:**
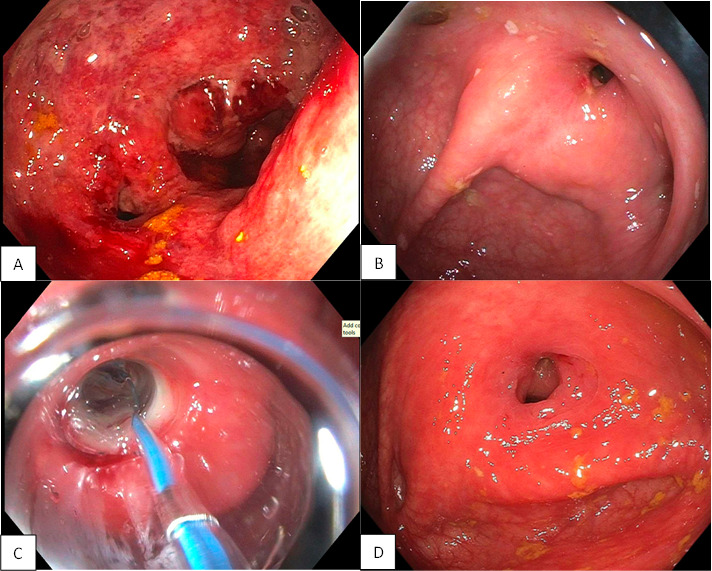
(A) Colonoscopy at time of diagnosis: Diffuse severe inflammation with ulceration, friability, and edema in the ascending colon with a high-grade stricture unable to be traversed. (B) Colonoscopy at 6 months: near complete resolution of inflammation and ulceration with persistent high-grade ascending colon stricture unable to be traversed. (C) Colonoscopy at 6 months: stricture dilation to 9 mm. (D) Colonoscopy at 1 year: complete resolution of inflammation and ulceration, stricture narrow but able to be traversed with endoscopically normal terminal ileum and cecum visualized.

A computed tomography (CT) chest with contrast identified fibrotic and consolidative changes in the bilateral upper lobes with volume loss, cavitation in the left apex, and innumerable tree-in-bud centrilobular nodules in both lung bases. A CT enterography showed ileocecal circumferential wall thickening and mucosal hyperemia, peri-cecal and peri-enteric fatty inflammatory changes, and prominent regional mesenteric lymph nodes. Findings were highly suspicious for pulmonary TB with transbronchial spread and ileocecal involvement. A colonoscopy revealed diffuse severe inflammation with ulceration, friability, and edema in the ascending colon with a high-grade stricture unable to be traversed (Fig. 1).

**Fig 2:**
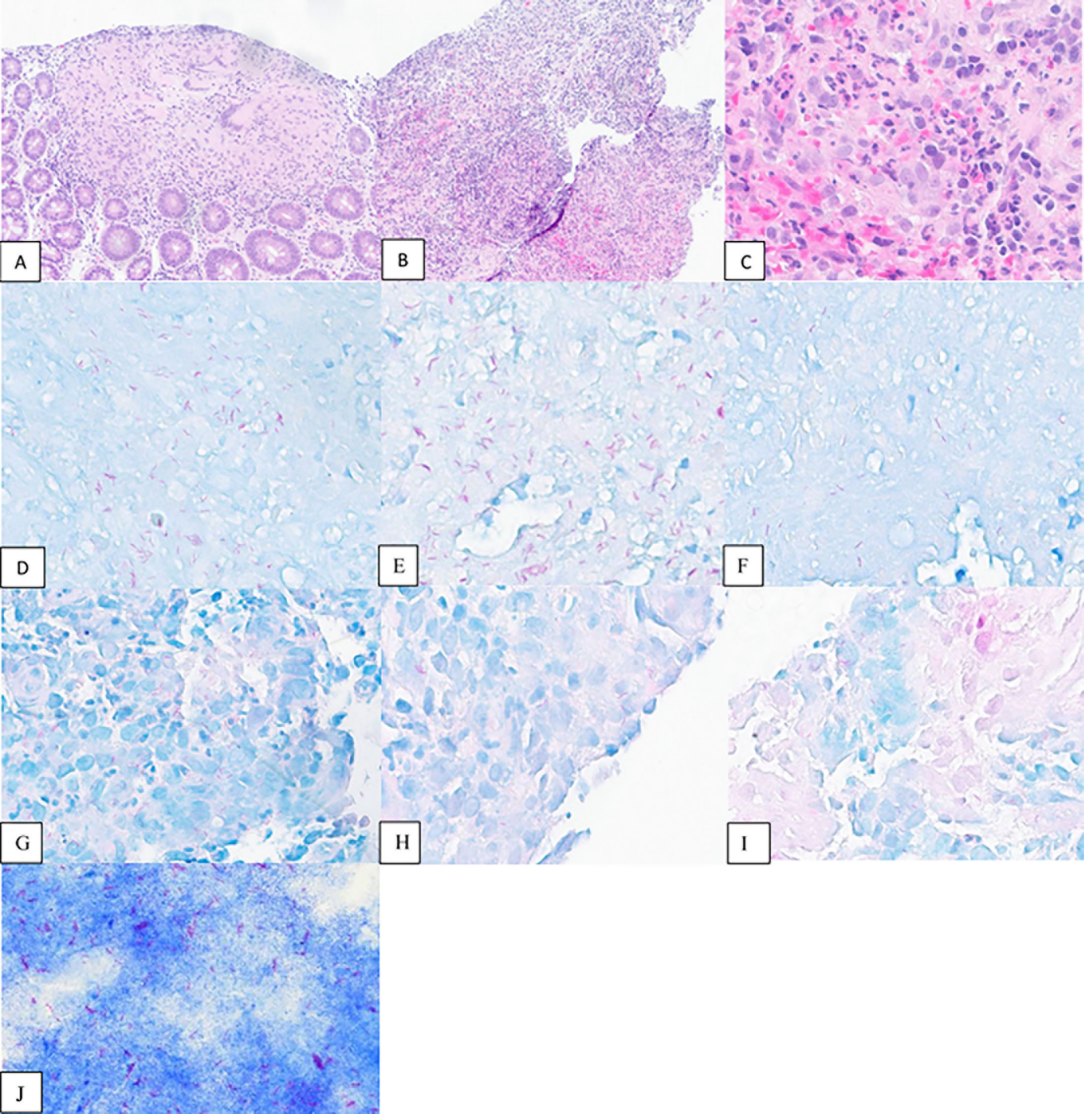
(A) Non-caseating granuloma (ascending colon, 100× magnification). (B) Granulation tissue (ascending colon, 100× magnification). (C) Active inflammation (ascending colon, 400×). (D–F) AFB stain (ascending colon, 400×). (G–I) Fite stain (ascending colon, 400×). (J) Touch prep with AFB stain (100×).

Multiple biopsies were taken from the right colon and showed AFB and Fite-positively staining bacilli with non-caseating granuloma (Fig. 2). Auramine/rhodamine stain performed on tissue showed acid-fast bacilli. AFB were seen in the broth from sputum culture in 7H9/MGIT after 7 days. Real-time PCR (Xpert MTB/RIF, Cepheid) of sputum was performed and detected MTB DNA. Sputum and processed sputum sediment specimens were processed in-house at SBUH, per manufacturer instructions. Tissue from the GI tract was tested by MTB/RIF PCR at the reference laboratory (ARUP) per off-label validation and was positive for MTB, concurrent with in-house testing and identification. An additional culture isolate was sent to ARUP for final identification and was confirmed as MTB by MALDI-ToF MS-MS (Bruker MALDI-ToF Siris RUO library). A culture isolate sent to the New York State Department of Health (Wadsworth Center) revealed the organism to be MTB Lineage 4 (L4) and was predicted to be pan-susceptible by whole genome sequencing.

The patient was started on antitubercular medications isoniazid (300 mg), rifampin (600 mg), pyrazinamide (1,500 mg/kg), and ethambutol (1,200 mg/kg), which was briefly discontinued after approximately 2 weeks due to a self-limited transaminitis. During the same admission, he also developed a partial small bowel obstruction, which resolved with conservative management. The patient was discharged after a 6-week admission with stable liver function tests.

An interval colonoscopy performed at 6 months showed near complete resolution of inflammation and ulceration, with a persistent high-grade stricture dilated to 9 mm. A subsequent interval colonoscopy at 1 year showed complete resolution of prior inflammation and ulceration; while the stricture was still present, it was traversed and showed a normal terminal ileum and cecum (Fig. 1). Tissue biopsies at both 6 month and 1 year intervals were negative for microorganisms on AFB and Fite stains and showed no granulomatous inflammation. The patient has continued to remain asymptomatic with a plan for repeat colonoscopy within the next 2 years. Repeat blood work showed return to normal ranges for hemoglobin, mean corpuscular volume, and platelet count.

## DISCUSSION

Infections with MTB result in the clinical disease known as TB. The mycobacteria that comprise this species complex include *Mycobacterium tuberculosis*, *Mycobacterium bovis*, *Mycobacterium bovis* BCG*, Mycobacterium africanum*, and *Mycobacterium microti* (7). GI TB is a rare extrapulmonary manifestation. Other common sites of extrapulmonary disease include the lymphatics, bones, and the nervous system (8). In high-income countries, GI TB is seen most commonly in immigrants and is associated with HIV/AIDS and immunosuppressive treatments. National TB surveillance data show a higher incidence in the younger population, females, Asians, and Blacks (8). The presented case is a rare example of an immunocompetent patient with extensive GI involvement, although exact numbers are difficult to ascribe. Current data suggest that the majority of extrapulmonary TB cases occur in immunocompromised patients, with ~50% or more decedents in an autopsy-based study concomitantly having HIV coinfection (9).

Extrapulmonary TB is thought to, in most cases, manifest following initial pulmonary infection but has been noted to not always be present with GI TB (3). Spread to other organ systems can be through lymphatics, the bloodstream, contiguous spread, or through ingestion of infected sputum (10). Multiple mechanisms have been proposed for the dissemination process: as MTB is an intracellular pathogen, transportation from the lungs may occur through alveolar macrophages and into blood or lymphatics; similarly, dendritic cells may act as an avenue of transport to lymph nodes; another proposed mechanism involves direct infection of epithelial cells and translocation across the barrier (10). Microfold cells could also be involved with transportation into the interstitium (10).

The ileocecal region is the most common site of GI TB manifestations (11). It can also affect sites as varied as the esophagus, gastric, small intestine, large intestine, rectum, anus, and peritoneal regions (6). Associated symptoms include abdominal pain, night sweats, fevers, diarrhea, and constipation. Due to the chronicity of the disease, diagnosis may result in a delay of patients seeking medical care through initial misdiagnosis (6). TB enteritis can be classified as ulcerative, hypertrophic, and ulcerohypertrophic, with the ulcerative type being the most common (4). Transverse ulcers are identified circumferentially in the jejunum, ileum, and cecum. Hypertrophic TB enteritis presents as an inflammatory pseudotumor with scarring and fibrosis (4). Ulcerohypertrophic TB contains both an inflammatory pseudotumor and thickening and ulceration of the intestinal wall (6). The complications of all three types include perforation, bleeding, fistulas, obstruction, and stricture formation (6). Microscopic evaluation can show caseating and noncaseating granulomas in the submucosa and serosa, and special stains for AFB and Fite can reveal the organisms.

Certain findings may help differentiate GI TB from Crohn’s. The presence of pulmonary symptoms and a shorter history (less than 6 months) favor GI TB, whereas chronic diarrhea, hematochezia, perianal symptoms, and extraintestinal manifestations, such as arthritis, erythema nodosum, uveitis, and liver involvement in the form of primary sclerosing cholangitis, favor Crohn’s (5). In terms of imaging findings, necrotic lymph nodes, a patulous ileocecal valve, short strictures, asymmetric mural thickening, and pulmonary lesions favor GI TB, whereas mural stratification, fibrofatty proliferation, skip lesions, and long segment involvement (>3 cm) favor Crohn’s (5). On endoscopy, a patulous ileocecal valve and circumferential or transverse ulcers favor GI TB, whereas aphthous ulcers, longitudinal ulcers, serpiginous ulcers, cobblestoning, and skip lesions favor Crohn’s (5). On histopathology, a large granuloma, confluent granuloma, caseating granuloma, or ulcer lined by histiocytes favor GI TB, whereas colitis, microgranuloma, and sparse granuloma favor Crohn’s disease (5).

The clinical presentation of GI TB can be asymptomatic or includes fever, anorexia, weight loss, nausea/vomiting, abdominal pain, diarrhea, and constipation (5). It is unlikely for there to be hematemesis and melena (5). Physical examination may reveal signs such as pallor, abdominal distention, ascites, hepatomegaly, splenomegaly, rectal bleeding, and abdominal lumps, depending on the structures involved in the GI tract (5). Laboratory tests may reveal nonspecific results such as mild anemia with a normal white blood cell count, and elevated acute phase reactants, such as C-reactive protein and erythrocyte sedimentation rate. Interferon gamma release assays may be performed; however, in many cases of GI TB, they can be negative. In addition, the tuberculin skin test may be falsely positive in individuals with administration of the BCG vaccine (5). Direct smear of the ascitic fluid in cases of peritoneal TB for bacteria is of low diagnostic value due to the pauci-bacillary nature of ascitic fluid (4).

Culture from biopsy specimens, ascitic fluid, or fecal samples is considered the gold standard for the diagnosis of GI TB; however, it presents significant challenges due to the slow-growing nature of the bacteria and the paucibacillary nature of the disease (12). Mycobacterial culture using traditional culture methods, such as Löwenstein-Jensen medium and the Middlebrook 7H10 and 7H11 agar, may take up to 8 weeks, while liquid media, such as Middlebrook 7H9 broth, and specialized media for drug susceptibility testing (such as the BACTEC MGIT 960 System) have a shorter turn-around time and higher sensitivity (12). The culture positivity rates on Löwenstein-Jensen medium vary from 7% to 48% and that of MGIT from 15% to 79% (13). Culture can also be used for species identification, drug susceptibility testing, and genotyping (12). Despite its diagnostic utility, culture positivity in GI TB is relatively low (12). As a result, clinicians often rely on a combination of culture, histopathology, molecular diagnostics, such as PCR, and clinical response to anti-tubercular therapy for definitive diagnosis (12). Adenosine deaminase level in ascitic fluid is also a potentially useful marker which can assist with the diagnosis of TB in the peritoneum, although it can also be elevated in cirrhosis (3). Advancements in liquid culture systems and molecular techniques continue to enhance diagnostic yield, yet culture remains an essential tool in confirming the presence of viable *M. tuberculosis* and conducting drug susceptibility testing to guide appropriate treatment strategies (12).

PCR and multiplex PCR are helpful technologies that aid in detecting cases of GI TB (12). Although not routinely validated, fresh tissue biopsies which are positive by AFB stains (and other stains like Fite, etc.), are of highest diagnostic yield for testing by nucleic acid amplification tests. The Xpert MTB/RIF (Cepheid) assay is a common method for molecular TB detection widely used in the United States for sputum specimens, but validation of other specimen types does require extensive work, which most sentinel laboratories are not capable of. Formalin-fixed, paraffin-embedded tissue may also be viable as a specimen type but could have increased risk of false-negative results due to fragmentation of DNA upon processing. These assays are routinely available at reference and public health laboratories.

Regarding imaging, the CT scan is the modality of choice used to detect GI TB (6). Imaging characteristics of GI TB on CT scan include: asymmetric wall thickening of the terminal ileum, cecum, or ileocecal valves and necrotic lymphadenopathy; a small and irregular patulous ileocecal valve due to fibrosis and/or stenosis (5). Endoscopically, GI TB may show shallow ulcers in the esophagus and may show a patulous ileocecal valve and circumferential or transverse ulcers on colonoscopy (3, 8).

In our case, the organism was revealed to be MTB Lineage 4, one of the main phylogenetic lineages of *M. tuberculosis* which represents one of the most widely distributed lineages, with high prevalence in all inhabited continents (14). Lineage 4 is responsible for the highest burden of TB globally (14). In Ecuador, Lineage 4 is the predominant strain (14). It is typically responsive to first-line anti-tuberculosis drugs; however, drug resistance can still occur (14). Therefore, it is important to conduct drug resistance testing (which can be done using molecular/genotypic testing or traditional culture-based phenotypic drug susceptibility testing).

The mortality of GI TB is generally low, if the diagnosis is rendered early and prompt anti-tubercular therapy is initiated; however, it is not zero, and there have been several case reports in the literature which report cases of fatal GI TB with autopsy findings (9). In the case of intestinal TB, bowel obstruction, perforation with resulting peritonitis, and gastrointestinal bleeding can all be fatal if not treated appropriately (usually with surgery). Small intestinal TB with strictures usually responds to anti-tubercular therapy without the need for surgery unless the strictures are long (greater than 12 cm) with multiple areas of involvement (15). In cases of GI TB with colonic involvement, mortality is generally very low (15). In addition to pharmacologic therapy, treatment of colonic GI TB with stricture may include endoscopic dilatation or surgery, especially when there are cases which are refractory to drugs, perforation, obstruction, and/or malignancy (13, 16). Long-term sequelae of GI TB include strictures in the GI tract, calcifications, and hypertension due to compression of venous structures by enlarged lymph nodes or organs, such as the liver.

In the HIV-positive population, an important differential diagnosis is GI *Mycobacterium avium* complex (MAC) infection, which refers to *M. avium* and *M. intracellulare* (difficult to differentiate, therefore collectively referred to as MAC). MAC organisms are ubiquitous, and sources include environmental water supplies that have been aerosolized, soil, birds, and farm animals (17). The infection is usually pulmonary; however, in immunocompromised patients, it can cause enlargement of the liver, spleen, and spread to the joints, lymph nodes, central nervous system, and bone marrow (17). Biopsy of the liver reveals well-formed granulomas composed of histiocytes (possibly large and foamy in immunocompromised patients) and rare multinucleated giant cells (17). The organisms, usually abundant, exhibit positivity on AFB and Periodic acid–Schiff stains (17).

### Conclusion

We have presented a case of a 29-year-old male who presented with a 2-month history of abdominal pain, which was later diagnosed as GI TB after the performance of biopsies, microscopic examination, and PCR. The patient was treated with anti-tubercular therapy, timely initiation of which is essential and which usually leads to significant clinical improvement.

This case highlights the need for awareness and vigilance among healthcare providers to ensure prompt diagnosis and appropriate treatment of GI TB. Clinicians should maintain a high index of suspicion for GI TB, especially in patients with a history of TB exposure or residence in high-prevalence areas presenting with unexplained gastrointestinal symptoms. Further research and continuous medical education are necessary to enhance the understanding of GI TB and improve diagnostic techniques and treatment protocols for GI TB.

## References

[1] Dheda K, Barry CE, Maartens G. 2016. Tuberculosis. Lancet 387:1211–1226. doi:10.1016/S0140-6736(15)00151-8 26377143 PMC11268880

[2] Golden MP, Vikram HR. 2005. Extrapulmonary tuberculosis: an overview. Am Fam Physician 72:1761–1768. 16300038

[3] Eraksoy H. 2021. Gastrointestinal and abdominal tuberculosis. Gastroenterol Clin North Am 50:341–360. doi:10.1016/j.gtc.2021.02.004 34024445

[4] Tobin EH, Khatri AM. 2025. Abdominal tuberculosis. StatPearls. 32310575

[5] Choudhury A, Dhillon J, Sekar A, Gupta P, Singh H, Sharma V. 2023. Differentiating gastrointestinal tuberculosis and Crohn’s disease- a comprehensive review. BMC Gastroenterol 23:246. doi:10.1186/s12876-023-02887-0 37468869 PMC10354965

[6] Malikowski T, Mahmood M, Smyrk T, Raffals L, Nehra V. 2018. Tuberculosis of the gastrointestinal tract and associated viscera. J Clin Tuberc Other Mycobact Dis 12:1–8. doi:10.1016/j.jctube.2018.04.003 31720391 PMC6830173

[7] Zhang H, Liu M, Fan W, Sun S, Fan X. 2022. The impact of Mycobacterium tuberculosis complex in the environment on one health approach. Front Public Health 10:994745. doi:10.3389/fpubh.2022.994745 36159313 PMC9489838

[8] Al-Zanbagi AB, Shariff MK. 2021. Gastrointestinal tuberculosis: a systematic review of epidemiology, presentation, diagnosis and treatment. Saudi J Gastroenterol 27:261–274. doi:10.4103/sjg.sjg_148_21 34213424 PMC8555774

[9] Mantilla JC, Chaves JJ, Africano-Lopez F, Blanco-Barrera N, Mantilla MJ. 2023. Gastrointestinal tuberculosis: an autopsy-based study. Infect Med (Beijing) 2:122–127. doi:10.1016/j.imj.2023.04.007 38077832 PMC10699657

[10] Moule MG, Cirillo JD. 2020. Mycobacterium tuberculosis dissemination plays a critical role in pathogenesis. Front Cell Infect Microbiol 10:65. doi:10.3389/fcimb.2020.00065 32161724 PMC7053427

[11] Choi EH, Coyle WJ. 2016. Gastrointestinal tuberculosis. Microbiol Spectr 4. doi:10.1128/microbiolspec.TNMI7-0014-2016 28084201

[12] Sharma M, Sharma K. 2022. Microbiological diagnosis of gastrointestinal tuberculosis. In Sharma V (ed), Tuberculosis of the gastrointestinal system, 1st ed. Springer, Singapore.

[13] Kedia S, Vineet A. 2022. Intestinal tuberculosis: an overview. In Sharma V (ed), Tuberculosis of the gastrointestinal system, 1st ed. Springer, Singapore.

[14] Garzon-Chavez D, Garcia-Bereguiain MA, Mora-Pinargote C, Granda-Pardo JC, Leon-Benitez M, Franco-Sotomayor G, Trueba G, de Waard JH. 2020. Population structure and genetic diversity of Mycobacterium tuberculosis in Ecuador. Sci Rep 10:6237. doi:10.1038/s41598-020-62824-z 32277077 PMC7148308

[15] Nath P. 2022. Epidemiology of gastrointestinal tuberculosis. In Sharma V (ed), Tuberculosis of the gastrointestinal system, 1st ed. Springer, Singapore.

[16] Orellana M, Vegas L, Cáceres A, Villarroel M, Soto P. 2022. Obstructive intestinal tuberculosis managed surgically: a case report and literature review. Int J Surg Open 41:100457. doi:10.1016/j.ijso.2022.100457

[17] Kanel G. 2017. Chapter 5. Non-viral infectious diseases. In Pathology of liver diseases, 1st ed. John Wiley & Sons, Incorporated.

